# Association between Tracheostomy Timing and Clinical Outcomes in Critically Ill COVID19 Patients

**DOI:** 10.22038/ijorl.2025.86206.3941

**Published:** 2025

**Authors:** Nikzad Shahidi, Ata Mahmoudpour, Mehrdad Shahidi, Hesam Shahmohammadi

**Affiliations:** 1 *Department of Otorhinolaryngology, Faculty of Medicine, Tabriz University of Medical Sciences, Tabriz, Iran.*; 2 *Department of Anesthesiology, Faculty of Medicine, Tabriz University of Medical Sciences, Tabriz, Iran.*; 3 *Department of Clinical Pharmacy, Faculty of Pharmacy, Tabriz University of Medical Sciences, Tabriz, Iran.*; 4 *Faculty of Medicine, Tabriz University of Medical Sciences, Tabriz, Iran.*

**Keywords:** COVID19, Tracheostomy, Intubation, Mechanical ventilation, Intensive care unit

## Abstract

**Introduction:**

The COVID19 pandemic has posed one of the greatest challenges to healthcare systems worldwide. Tracheostomy is often required in critically ill patients with COVID19 who require prolonged mechanical ventilation and frequent airway clearance. Determining the optimal timing of tracheostomy in these patients, particularly after endotracheal intubation, remains clinically complex and controversial.

**Materials and Methods:**

This retrospective study included COVID19–positive patients (confirmed by PCR) admitted to a referral hospital in Northwest Iran who underwent tracheostomy during their ICU stay. Patients were stratified into early and late tracheostomy groups based on the interval between intubation and tracheostomy (<14 days vs. ≥14 days). Demographic data, duration of mechanical ventilation before and after tracheostomy, and survival rates were analyzed.

**Results:**

A total of 62 patients were evaluated. Fourteen patients (22.6%) underwent early tracheostomy, while fortyeight patients (77.4%) underwent late tracheostomy. The mean duration of mechanical ventilation after tracheostomy was 28.57 days in the early group and 30 days in the late group. The overall duration of mechanical ventilation was significantly shorter in the early group compared with the late group (39.36 vs. 58.42 days). Survival rates were 57.1% in the early group and 39.6% in the late group.

**Conclusion:**

Early tracheostomy-performed within the first 14 days following intubation-significantly decreases the total duration of mechanical ventilation in critically ill COVID19 patients. However, tracheostomy timing does not influence the duration of ventilation after tracheostomy or overall patient survival.

## Introduction

The coronavirus disease 2019 (COVID19) pandemic has resulted in extensive global mortality and placed tremendous strain on healthcare systems due to its rapid transmission and severe complications (1,2). The unprecedented nature of the outbreak initially limited available research, prompting a worldwide effort to better characterize the disease and leading to continual updates in diagnostic and therapeutic protocols as evidence accumulated (2).

A major aspect of managing severe COVID19 has been the provision of intensive care. Epidemiological data indicate that up to 20 % of infected patients require hospitalization, and approximately 25 % of these patients need intensive care unit (ICU) admission, with a considerable portion requiring invasive mechanical ventilation (3).

Indications for endotracheal intubation include respiratory deterioration despite noninvasive ventilation or the development of other complications. In patients needing prolonged ventilatory support, tracheostomy may ultimately be required. Tracheostomy, the direct establishment of an airway through the trachea, can be performed via open or percutaneous techniques; the percutaneous approach, being less invasive and faster, may reduce exposure risk for healthcare workers (4).

Determining the optimal timing and approach for tracheostomy in COVID19 patients is complex, as the procedure generates aerosols that can increase viral transmission risk to frontline staff. Thus, multidisciplinary decisionmaking is essential to balance patient benefit against occupational safety. While delaying tracheostomy may mitigate transmission risk, it can also negatively influence clinical outcomes, making timing a pivotal issue in the management of these patients (5). 

Globally, data from the United States, China, the United Arab Emirates, Italy, and other countries have described the frequency, timing, and outcomes of tracheostomy in COVID19 patients(6) Most studies categorize early tracheostomy as performed within 10–15 days after intubation and late tracheostomy thereafter (7). Evidence suggests that early tracheostomy can shorten the duration of mechanical ventilation and ICU stay, although survival benefits have been inconsistent. Both open and percutaneous techniques have been shown to be safe for patients and staff when appropriate protective protocols are implemented (8).

Despite these international insights, there remains a significant knowledge gap concerning tracheostomy practice in Iranian COVID19 patients (9). To date, no comprehensive studies have investigated the local epidemiology, timing, and outcomes of tracheostomy procedures, limiting comparisons with global standards and the adaptation of evidencebased guidelines to local contexts (10). To the best of our knowledge, this is the first comprehensive study examining the frequency, timing, and clinical outcomes of tracheostomy among critically ill COVID19 patients admitted to a major referral hospital in Northwest Iran (11).

Unlike previous studies conducted abroad or through limited case series, the present investigation draws from highvolume, realworld clinical data in Iran (12,13) This allows assessment of patient outcomes together with evaluation of the protective measures applied for healthcare worker safety during tracheostomy. Notably, in 2021, Dr. Philip Staibano and colleagues published a systematic review and metaanalysis assessing the association between tracheostomy in COVID19 patients and the risk of SARSCoV2 transmission to healthcare workers (14). Their analysis pooled multiple studies, demonstrating a mean patient age of 60.7 years, with 73.8 % male and 26.2 % female. Three studies reported incidents of healthcare worker infection linked to tracheostomy procedures. Moreover, early tracheostomy was associated with discharge from the ICU approximately 6.17 days earlier, without significant impact on ventilator weaning, decannulation, operative complications, or mortality.

Building upon this context, the present study aims to determine the frequency and timing of tracheostomy in ICUadmitted COVID19 patients at our referral center in Northwest Iran and to evaluate its impact on the duration of mechanical ventilation, ICU stay, and mortality. Through a comparative analysis of early versus late tracheostomy aligned with both international and local practices, this research addresses a crucial gap in Iran’s literature and contributes locally relevant evidence to inform clinical decisionmaking and health policy during current and future critical care crises.

## Materials and Methods

This crosssectional study was conducted over a 1.5year period at *Imam Reza Hospital*, a major tertiary referral center located in Northwest Iran. The study period extended from May 1, 2023 to September 30, 2024. All patients aged 18 years and older who tested positive for COVID19 by polymerase chain reaction (PCR), were admitted to the intensive care unit (ICU) of Imam Reza Hospital in Tabriz, and underwent tracheostomy during hospitalization were eligible for inclusion.

Inclusion criteria were established to ensure a uniform and representative study population: (1) confirmed positive PCR test for COVID19 on initial admission, (2) ICU admission at Imam Reza Hospital, (3) age > 18 years, and (4) performance of tracheostomy during the hospital stay. Patients were excluded if they met any of the following conditions: negative PCR test for COVID19 at any point during admission, absence of tracheostomy, age < 18 years, incomplete medical records or missing data in the hospital archives, transfer to another institution before tracheostomy, or tracheostomy performed at a different hospital prior to transfer. To maintain data reliability, patients with asthma or other underlying pulmonary diseases were also excluded. Eligible patients were categorized according to the interval between intubation and tracheostomy into early tracheostomy (< 14 days after intubation) and late tracheostomy (≥ 14 days after intubation) groups. For each group, the following variables were recorded: mean patient age, sex distribution (male/female), and blood type. The prevalence of underlying comorbidities and the type of tracheostomy technique (open versus percutaneous) were also documented. 

Additionally, outcomes were assessed based on whether patients were discharged with a tracheostomy cannula, underwent decannulation, or died during their hospital course. Data were collected retrospectively using a structured extraction form developed by the research team. This tool was designed to systematically retrieve clinical and demographic information from electronic medical records and archived files of ICU patients at Imam Reza Hospital (Tabriz, Iran).

Continuous variables were first tested for normality using the Kolmogorov–Smirnov test. Depending on the distribution pattern, appropriate statistical procedures were applied. For normally distributed variables, parametric tests were used, while nonparametric tests were applied to nonnormal data. The twosample ttest assessed differences in the total duration of mechanical ventilation between early and late tracheostomy groups. When data were not normally distributed, the Mann–Whitney U test served as the nonparametric alternative.

Categorical variables were analyzed using the chisquare test, particularly to examine the relationship between tracheostomy timing and patient discharge status as well as to compare the distribution of blood groups among patients with that of the general population. All analyses were performed using R software (version 4.3.1), with the statistical significance level set at p < 0.05. Graphs and tables were generated using Microsoft Excel 2019.

Survival rate was defined as the proportion of individuals alive after a specified period (e.g., 30 days, 1 year, or 5 years). The basic survival rate formula used was:

$\mathrm{Survival Rate}(\%)=\frac{\mathrm{Number of patients alive at end of period}}{\mathrm{Total number of patients at start of period}}\times 100$

For comprehensive assessment, advanced survival analysis was performed using the Kaplan–Meier estimator, which accounts for censored data such as patients lost to followup or those still alive at the study endpoint. 

This estimator calculates the probability of survival at successive intervals and multiplies these probabilities cumulatively to construct a survival curve. When the influence of multiple covariates on survival was explored, the Cox proportional hazards model was employed to provide a multivariate analysis of survival determinants.

This study was reviewed and approved by the Ethics Committee of Tabriz  University  of  Medical Sciences, under the protocol code IR. TBZMED. REC. 1402. 034. All legal, professional, and ethical responsibilities rest with the principal investigator and the research team. No additional costs were imposed on participating patients at any point related to treatment or data inclusion. Given the retrospective nature of this study, all patient data were kept strictly confidential, and identifiers were anonymized prior to analysis.

## Results

A total of 62 patients met the inclusion criteria and were enrolled in the study. All patients underwent tracheostomy following endotracheal intubation, with an overall mean interval of 22.72 ± 9.47 days between the two procedures. Among these, 14 patients (22.6%) received early tracheostomy, defined as being performed within the first 14 days after intubation. The mean duration from intubation to tracheostomy in the early group was 11.8 ± 3.04 days. The remaining 48 patients (77.4%) underwent late tracheostomy, defined as occurring more than 14 days after intubation, with a mean interval of 22.72 ± 9.48 days between intubation and tracheostomy. The mean age of all participants was 59.95 ± 15.37 years. Patients in the early group had a mean age of 63.21 ± 16.23 years, while those in the late group had a mean age of 59.15 ± 17.15 years. Of the 62 patients, 27 (43.5%) were female and 35 (56.5%) were male. Within the early tracheostomy group, 4 (28.6%) were female and 10 (71.4%) were male; in contrast, the late group comprised 23 females (47.9%) and 25 males (52.1%) (Table 1).

Regarding blood type distribution, 24  patients (39%) had blood group A, 8 (13%) group B, 8 (13%) group AB, and 22 (34%) group O. A chisquare test comparing this distribution with that of the general population produced a pvalue = 0.1, indicating that at a significance level of 0.05, there was no statistically significant difference between the blood group frequencies of the study cohort and the general population. To improve clinical comparability between the early and late tracheostomy groups, the expanded baseline characteristics table incorporated two additional domains: comorbidities and presenting symptoms at hospital admission. The documented comorbidities included hypertension, diabetes mellitus, cardiovascular disease, malignancy, and chronic kidney disease—conditions known to influence the risk profile and prognosis of critically ill patients, particularly those undergoing tracheostomy. Reporting these comorbidities for both groups enables a clearer understanding of baseline health status and assists in controlling for potential confounding variables during outcome analysis. The section on presenting symptoms, such as fever, cough, dyspnea, and fatigue, describes the initial clinical manifestations observed upon admission. Inclusion of these parameters allows comparison of initial disease severity between groups and ensures that any subsequent differences in outcomes are less likely attributable to disparities in baseline clinical presentation (Table 1).

**Table 1:** Baseline Characteristics of Patients in Early and Late Tracheostomy Groups

**Variable**	**Total (n=62)**	**Early Group (n=14)**	**Late Group (n=48)**	**P Value**
**Interval, intubation to tracheostomy(**days)	22.72 ± 9.47	11.8 ± 3.04	25.2 ± 7.92 *	˂0.001 *
**Mean age (years)**	59.95 ± 15.37	63.21 ± 16.23	59.15 ± 17.15	0.410 *
**Sex**				
Female, n (%)	27 (43.5%)	4 (28.6%)	23 (47.9%)	0.210 **
Male, n (%)	35 (56.5%)	10 (71.4%)	25 (52.1%)	
**Blood Group, n (%)**				
A	24 (39%)	5 (35.7%)	19 (39.6%)	0.933 **
B	8 (13%)	2 (14.3%)	6 (12.5%)	
AB	8 (13%)	1 (7.1%)	7 (14.6%)	
O	22 (34%)	6 (42.9%)	16 (33.3%)	
**Comorbidities, n (%)**				
Hypertension	27 (43.5%)	6 (42.9%)	21 (43.8%)	0.989 **
Diabetes mellitus	21 (33.9%)	6 (42.9%)	15 (31.3%)	0.406 **
Cardiovascular disease	15 (24.2%)	4 (28.6%)	11 (22.9%)	0.722 **
Malignancy	7 (11.3%)	2 (14.3%)	5 (10.4%)	0.644 **
Chronic kidney disease	5 (8.1%)	1 (7.1%)	4 (8.3%)	0.872 **
**Presenting symptoms, n (%)**				
Fever	45 (72.6%)	10 (71.4%)	35 (72.9%)	0.909 **
Cough	39 (62.9%)	9 (64.3%)	30 (62.5%)	0.902 **
Dyspnea	53 (85.5%)	13 (92.9%)	40 (83.3%)	0.380 **
Fatigue	36 (58.1%)	8 (57.1%)	28 (58.3%)	0.943 **
* Independent t-test ; ** Chi-square				

Among the 62 patients included in the study, a total of 41 tracheostomies (66.1%) were performed using the open surgical technique, while 21 tracheostomies (33.9%) were performed by the percutaneous method. In the early tracheostomy group, 11 procedures were completed as open tracheostomies and 3 using the percutaneous approach. In contrast, the late tracheostomy group included 30 open and 18 percutaneous procedures. 

Of the 41 patients who underwent open tracheostomy, 20 (48.8%) survived and were discharged, whereas 21 (51.2%) died during their hospital stay. Among the 21 patients who received percutaneous tracheostomy, 7 (33.33%) survived and were discharged, and 14 (66.67%) did not survive. Thus, the survival rate was 48.8% in the open tracheostomy group compared with 33.33% in the percutaneous group.

Patients required mechanical ventilation for an overall mean duration of 29.67 ± 22.18 days after tracheostomy. The early tracheostomy group remained on mechanical ventilation for an average of 28.57 ± 16.67 days, while the late group required ventilation for 30 ± 23.69 days following tracheostomy. Normality of the posttracheostomy ventilation duration was verified using the Kolmogorov–Smirnov test, which yielded a pvalue = 0.38, confirming that the data followed a normal distribution at the α = 0.05 significance level. 

Subsequently, a twosample ttest was employed to compare the duration of mechanical ventilation between the early and late tracheostomy groups. 

The resulting pvalue = 0.07 indicated no statistically significant difference between the two groups (p > 0.05). Therefore, the observed minor difference in mean posttracheostomy ventilation duration between the early and late groups was not statistically meaningful.

The total duration of mechanical ventilation-defined as the full period from intubation until successful weaning—was 52.40 ± 25.41 days across all patients. 

**Fig 1:**
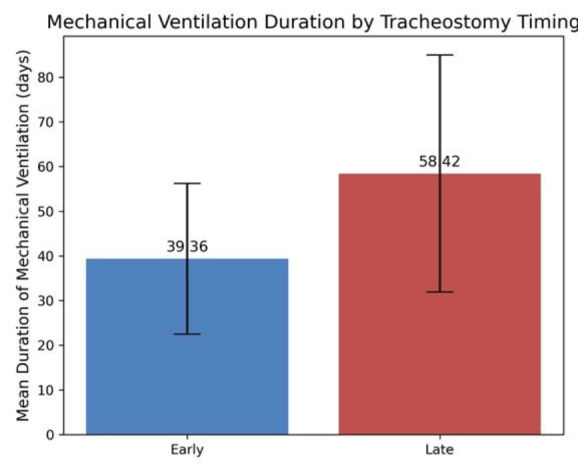
Comparison of Mechanical Ventilation Duration Between Early and Late Tracheostomy Groups

The early tracheostomy group required mechanical ventilation for an average of 39.36 ± 16.86 days, whereas the late tracheostomy group had a markedly longer mean duration of 58.42 ± 26.52 days, a difference that reached statistical significance (p = 0.049) (Figure 1).

Notably, patients in the early tracheostomy group spent an average of approximately 19 days less on mechanical ventilation than those in the late tracheostomy group, highlighting a clinically meaningful reduction in total ventilation time. Among the 62 critically ill COVID19 patients who underwent tracheostomy, 27 (43.5%) were successfully discharged from the hospital. Of these, 2 patients were discharged without a tracheostomy cannula, while 25 patients were discharged with the cannula in situ. Unfortunately, 35 patients (56.5%) died during their hospital stay. For those who died, the mean time from tracheostomy to death was 21.88 ± 15.22 days.

**Fig 2:**
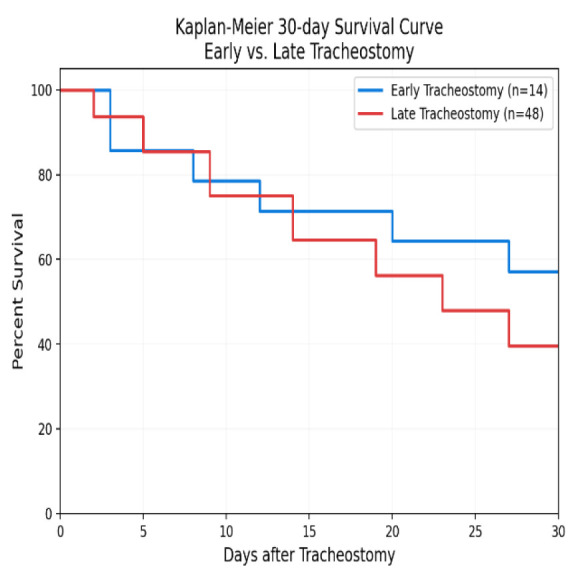
Kaplan-Meier Survival Curves for 30-day Outcome after Tracheostomy by Timing (Early vs. Late Groups)

In the early tracheostomy group, 8 patients (57.1%) survived and were discharged, whereas 6 patients (43.9%) did not survive. In contrast, in the late tracheostomy group, 19 patients (39.6%) were discharged alive and 29 patients (60.4%) died. Accordingly, the overall survival rate was 57.1% for the early group and 39.6% for the late group (Figure 2). A chisquare test was performed to examine the association between tracheostomy timing (early vs. late) and discharge status (alive vs. deceased). The resulting pvalue = 0.39 indicated no statistically significant association between tracheostomy timing and survival outcome. Thus, based on these findings, the timing of tracheostomy in critically ill COVID19 patients does not appear to significantly affect survival rates. However, as previously noted, the difference in overall duration of mechanical ventilation between the two groups-encompassing intubation to final weaning-was statistically significant, emphasizing the potential benefit of early tracheostomy for reducing total ventilatory support time.

Table 2 presents a comparative analysis of the most frequent posttracheostomy complications—namely bleeding, tube blockage, local infection, pneumothorax, subcutaneous emphysema, accidental decannulation, stomal granuloma, and tracheoesophageal fistula-in patients who underwent early versus late tracheostomy. The overall incidence of any complication was 21.4% in the early tracheostomy group and 31.3% in the late tracheostomy group, a difference that was not statistically significant (P = 0.454). Likewise, analysis of individual complications revealed that none occurred with significantly different frequencies between the two timing groups. These results indicate that performing tracheostomy within the first 14 days following intubation does not increase the risk of postoperative or procedurerelated complications when compared with later tracheostomy. Overall, early tracheostomy was safe and well tolerated, with a complication profile comparable to that of late tracheostomy in critically ill COVID19 patients.

**Table 2:** Comparison of Post-Tracheostomy Complications Between Early and Late Groups

**Complication**	**Total (n=62)**	**Early Group (n=14)**	**Late Group (n=48)**	P Value *
Bleeding	6 (9.7%)	1 (7.1%)	5 (10.4%)	0.684
Tracheostomy tube blockage	4 (6.5%)	1 (7.1%)	3 (6.3%)	0.903
Local infection	8 (12.9%)	2 (14.3%)	6 (12.5%)	0.857
Pneumothorax	3 (4.8%)	0 (0%)	3 (6.3%)	0.376
Subcutaneous emphysema	2 (3.2%)	0 (0%)	2 (4.2%)	0.487
Accidental decannulation	1 (1.6%)	0 (0%)	1 (2.1%)	0.653
Stomal granuloma	4 (6.5%)	1 (7.1%)	3 (6.3%)	0.903
Tracheoesophageal fistula	1 (1.6%)	0 (0%)	1 (2.1%)	0.653
Any complication	18 (29.0%)	3 (21.4%)	15 (31.3%)	0.454

*: Chi-square

## Discussion

This study analyzed 62 critically ill patients with COVID19 who underwent tracheostomy at our institution. In comparison, previous reports included 138 patients in *Kwak et al.*, 53 in *Chao et al.*, 21 in *Nadeem et al.*, and 153 in *Battaglini et al.*(10-13). In the current cohort, tracheostomy was performed at an average of 22.72 ± 9.47 days after intubation. This mean interval was 12.23 days in Kwak’s study, 19.7 days in Chao’s series, and 22.1 ± 7.5 days in Nadeem’s report. Such variability among studies underscores the ongoing debate regarding the optimal timing of tracheostomy in patients with COVID19—a subject complicated by limited prior data and the relative novelty of COVID19 as a criticalcare condition(10-12). For the purpose of this investigation, tracheostomy timing was classified as early if it occurred within 14 days of intubation and late if performed beyond this interval. This 14day cutoff has been widely used in comparable analyses, including Battaglini’s multicenter study. However, several authors such as Kwak and Pollock have employed a 10day threshold to define early intervention (11,13,15-19).

The mean age of our participants was 59.95 ± 15.37 years, which is consistent with findings from previous research: 58.1 ± 15.8 years in Kwak’s study, 62.0 ± 14.3 years in Chao’s, 60.7 years in Staibano’s metaanalysis, and 63.80 ± 9.24 years in Battaglini’s cohort. Across nearly all these studies, male predominance was observed, corroborating that older age and male sex are associated with more severe disease manifestations. This pattern was first described in a retrospective study by Xiaochen Li et al. (2020), which examined hospitalized COVID19 patients at Tangji Hospital, Wuhan, China, confirming higher morbidity and mortality among elderly male individuals(11, 14, 20). Blood group analysis further contextualizes disease characteristics. A 2021 retrospective study by Zhao et al. in Wuhan and Shenzhen, including 2,173 patients, demonstrated that individuals with blood group A had an increased susceptibility to COVID19, while those with blood group O showed a relatively reduced risk (21). In contrast, Kumar et al. (2021) evaluated 3,563 hospitalized patients in Georgia, reporting no significant differences in mechanical ventilation requirements or mortality across blood groups, though blood group AB showed a higher likelihood of tracheostomy during hospitalization (22). Collectively, these studies suggest that blood type may influence disease susceptibility, but does not affect major clinical outcomes among hospitalized COVID19 patients. Consistent with these observations, our analysis found no significant difference between the bloodgroup distribution of our cohort and that of the general population. Larger, multicenter studies are needed to more definitively delineate any potential correlation between ABO blood type and COVID19 outcomes. A systematic review and metaanalysis by Staibano et al. (2021) concluded that tracheostomy technique—open versus percutaneous—was not associated with differences in operative complications or mortality (15). Interestingly, across major centers analyzed in the latter review, percutaneous tracheostomy predominated; however, in our patient population, the incidence of open tracheostomy was approximately twice that of percutaneous tracheostomy. Moreover, the survival rate among patients who underwent open tracheostomy was about oneandahalf times higher than among those who received percutaneous procedures, although this observation may be influenced by numerous clinical and nonprocedural confounding variables that were beyond the scope of this study(7). Our analysis further revealed that posttracheostomy mechanical ventilation duration was comparable between the early and late tracheostomy groups, suggesting that timing did not influence ventilatory dependence after the procedure. This finding closely aligns with results from Chong et al.’s metaanalysis, which likewise reported no significant difference in posttracheostomy ventilation duration between groups. On average, patients in our study remained on mechanical ventilation approximately two weeks longer than the mean duration observed for 2,222 patients in Chong’s pooled analysis (7). It is noteworthy that, although posttracheostomy ventilation duration did not vary significantly, patients in the early group required markedly less total mechanical ventilation—a result consistent with Chong’s conclusion that early tracheostomy reduced overall ventilation time by approximately  8 days(7). Statistical evaluation in our cohort showed that tracheostomy timing did not significantly influence survival. This finding is supported by the results of Chung et al., who reported a 33% mortality rate among COVID19 patients undergoing tracheostomy (9). By comparison, the mortality rate in our study population at Imam Reza Hospital, Tabriz was more than 1.5 times higher. Similarly, Battaglini et al. reported that tracheostomy timing was not associated with survival outcomes, mirroring our own findings (13). Nevertheless, earlier studies such as Angel et al. had suggested a survival advantage in early tracheostomy, though subsequent larger cohort studies and metaanalyses indicated otherwise, confirming that timing of tracheostomy does not exert a significant influence on mortality among COVID19 patients (23-26)

*Strengths* *and limitations*

This study’s principal strengths lie in its comprehensive comparative evaluation of early versus late tracheostomy within a clearly defined cohort of critically ill COVID19 patients, coupled with an indepth assessment of survival outcomes and procedurerelated complications. The incorporation of stepwise Kaplan–Meier survival analysis and meticulous adjustment for baseline covariates strengthens the internal validity and reliability of the results. Nevertheless, certain limitations must be acknowledged. As a singlecenter investigation with a moderate sample size, the findings may be influenced by selection bias and might not be fully generalizable to broader populations or diverse clinical settings. Moreover, the observational design restricts the ability to establish causal relationships, and the potential impact of unmeasured confounding factors cannot be entirely excluded. Despite these constraints, the study provides important empirical evidence that enriches the ongoing international discourse regarding the optimal timing of tracheostomy in critically ill patients, offering both clinical insight and a foundation for future multicenter, randomized investigations.

## Conclusion

This study demonstrates that the timing of tracheostomy plays a clinically relevant role in determining the total duration of mechanical ventilation among critically ill patients with COVID19. Performing tracheostomy within the first 14 days after endotracheal intubation is associated with a significant reduction in overall ventilatory support time, suggesting potential benefits for earlier airway management in selected patients. In contrast, tracheostomy timing does not influence the length of postprocedural ventilation nor has any significant impact on survival outcomes. These findings emphasize the importance of individualized decisionmaking based on patient stability and institutional protocols rather than timing alone.

### Conflict of interest

 The authors declare that they have no conflicts of interest related to this study.
